# Classification of severe aortic stenosis and outcomes after aortic valve replacement

**DOI:** 10.1038/s41598-022-11491-3

**Published:** 2022-05-07

**Authors:** Yura Ahn, Se Jin Choi, Soyeoun Lim, Joon Bum Kim, Jong-Min Song, Duk-Hyun Kang, Jae-Kwan Song, Hwa Jung Kim, Joon-Won Kang, Dong Hyun Yang, Dae-Hee Kim, Hyun Jung Koo

**Affiliations:** 1 Department of Radiology and Research Institute of Radiology, Cardiac Imaging Center, Asan Medical Center, University of Ulsan College of Medicine, Olympic-ro, 388-1, Seoul, 05505 South Korea; 2 Department of Radiology, Ulsan University Hospital, University of Ulsan College of Medicine, Ulsan, South Korea; 3 Department of Cardiothoracic Surgery, Asan Medical Center, University of Ulsan College of Medicine, Seoul, South Korea; 4 Division of Cardiology, Cardiac Imaging Center, Asan Medical Center, University of Ulsan College of Medicine, Olympic-ro, 388-1, Seoul, 05505 South Korea; 5 Department of Clinical Epidemiology and Biostatistics, Asan Medical Center, University of Ulsan College of Medicine, Seoul, South Korea

**Keywords:** Cardiology, Diseases, Cardiovascular diseases

## Abstract

Aortic valve calcium scoring by cardiac computed tomographic (CT) has been recommended as an alternative to classify the AS (aortic stenosis) severity, but it is unclear that whether CT findings would have additional value to discriminate significant AS subtypes including high gradient severe AS, classic low-flow, low gradient (LF-LG) AS, paradoxical LF-LG AS, and moderate AS. In this study, we examined the preoperative clinical and cardiac CT findings of different subtypes of AS in patients with surgical aortic valve replacement (AVR) and evaluated the subtype classification as a factor affecting post-surgical outcomes. This study included 511 (66.9 ± 8.8 years, 55% men) consecutive patients with severe AS who underwent surgical AVR. Aortic valve area (AVA) was obtained by echocardiography (AVA_echo_) and by CT (AVA_CT_) using each modalities measurement of the left ventricular outflow tract. Patients with AS were classified as (1) high-gradient severe (n = 438), (2) classic LF-LG (n = 18), and (3) paradoxical LF-LG (n = 55) based on echocardiography. In all patients, 455 (89.0%) patients were categorized as severe AS according to the AVA_CT_. However, 56 patients were re-classified as moderate AS (43 [9.8%] high-gradient severe AS, 5 [27.8%] classic LF-LG AS, and 8 [14.5%] paradoxical LF-LG AS) by AVA_CT_. The classic LF-LG AS group presented larger AVA_CT_ and aortic annulus than those in high-gradient severe AS group and one third of them had AVA_CT_ ≥ 1.2 cm^2^. After multivariable adjustment, old age (hazard ratio [HR], 1.04, P = 0.049), high B-type natriuretic peptide (BNP) (HR, 1.005; P < 0.001), preoperative atrial fibrillation (HR, 2.75; P = 0.003), classic LF-LG AS (HR, 5.53, P = 0.004), and small aortic annulus on CT (HR, 0.57; P = 0.002) were independently associated with major adverse cardiac and cerebrovascular events (MACCE) after surgical AVR.

## Introduction

Evaluation of the severity of aortic valve stenosis (AS) is crucial for stratifying patient management and decision making for the timing of surgical intervention, especially in patients who suspected having significant AS. Echocardiography is the main modality to assess the degree of AS by measuring transaortic peak velocity, mean pressure gradient, and calculating aortic valve area (AVA). However, constant measurement using echocardiography is sometimes difficult, especially in low-flow, low-gradient (LF-LG) conditions. Classic LF-LG severe AS is defined by a small aortic valve (AV) area on echocardiography (AVA_echo_ < 1 cm^2^), a low mean pressure gradient (PG < 40 mmHg), and low flow (stroke volume [SV] < 35 mL/m^2^). The condition is characterised by low cardiac output due to a reduced left ventricular ejection fraction (LVEF < 50%)^1^. Conversely, LF-LG AS may occur despite preserved LVEF and is classified as paradoxical LF-LG AS. Since LF-LG AS presents fewer potential benefits from AV replacement (AVR) and considerable operation risks compared to true-severe AS, a classification for AS is important^2^.

Although the planimetry of the AVA using three-dimensional transoesophageal echocardiography has been reported to be more accurate than transthoracic echocardiography^3^, measurement issues still remain unresolved. Even in patients with normal systolic LV function, the grading of AS on echocardiography is inconsistent, and this is partly due to reduced SV^4,5^. In patients with low-flow state, AS severity may be underestimated due to lower mean PG, while incomplete opening of the AV may overestimate stenosis severity because of the reduced opening forces to the AV^6^. In patients with low-flow state, there can be a discrepancy between the effective orifice area and the PG. Moreover, the continuity equation assumes circular LV outflow tract (LVOT) which is elliptical shape, and echocardiography may underestimate LVOT. Additional diagnostic tests, dobutamine stress echocardiography (DSE)^7,8^ and AV calcium score (AVC) obtained by computed tomography (CT) scan^9,10^, have been used for the confirmation of severity and therapeutic guidance, and there is a chance that the patients with severe AS may be reclassified into the moderate range. However, reference standards used in these studies consisted of subjective assessment of the valve severity by cardiac surgeons and the AVC on CT images, which do not reflect hemodynamic severity.

Cardiac CT is recommended as an alternative to assess AS severity when DSE is inconclusive^11^. However, discrepancies have been reported between the measured AVA on cardiac CT (AVA_CT_) and AVA_echo_^12,13^. AVA_CT_ was significantly greater than the AVA_echo_ calculated by continuity equation, and suggested cut-off of AVA_CT_ for severe AS was < 1.2 cm^2^. Moreover, CT findings of different subtypes of AS and whether imaging has prognostic values remain undefined. Thus, we sought to (i) examine the preoperative CT characteristics of different subtypes of AS, and (ii) evaluate prognostic factors including CT findings affecting major adverse cardiovascular and cerebrovascular events (MACCE) after AVR.

## Results

### Patient characteristics

High-gradient severe AS (85.7% [438/511]) was most common among patients, followed by paradoxical LF-LG AS (10.8% [55/511]) and classic LF-LG AS (3.5% [18/511]) (Table 1). Half of the patients had tricuspid valves (48.1% [246/511]) and bicuspid valves were detected in the remaining patients. The median follow-up period for all patients was 4.12 (interquartile range [IQR], 3.19–5.50) years.

**Table 1:** Clinical and imaging characteristics of high-gradient severe, classic LF-LG, paradoxical LF-LG AS groups (n = 511).

Characteristic	High-gradient severe AS	Classic LF-LG AS	Paradoxical LF-LG AS	P-value
No. of patients (%)	438 (85.7)	18 (3.5)	55 (10.8)	
Age, years	66.8 ± 8.8	66.8 ± 6.2	67.3 ± 9.4	0.93
Male	236 (53.9)	13 (72.2)	31 (56.4)	0.30
BSA, m^2^	1.6 ± 0.2	1.7 ± 0.2	1.6 ± 0.2	0.56
Hypertension	231 (52.7)	12 (66.7)	30 (54.5)	0.50
Atrial fibrillation	65 (14.8)	4 (22.2)	4 (7.3)	0.20
PCI or CABG	94 (21.5)	9 (50.0)*	14 (25.5)	0.02
BNP, pg/mL	99.5 (43.0–280.5)	944.5 (304.8–3066.0)*	70.0 (35.0–190.0)	< 0.001
lnBNP	4.6 (3.8–5.6)	6.8 (5.7–8.0)*	4.2 (3.6–5.2)	< 0.001
**Echocardiography**				
LVEF, %	60.3 ± 10.0	36.0 ± 10.3*	62.6 ± 5.3	< 0.001
Peak velocity, m/s	5.2 ± 0.7	3.6 ± 0.5*	3.5 ± 0.5^†^	< 0.001
Peak PG, mmHg	108.5 ± 30.5	52.0 ± 13.1*	50.1 ± 16.0^†^	< 0.001
Mean PG, mmHg	66.5 ± 19.4	29.8 ± 7.9*	28.0 ± 9.5^†^	< 0.001
LVMI, g/m^2^	135.4 ± 35.8	149.6 ± 31.1	124.9 ± 36.2	0.03
AV VTI, cm	124.6 ± 26.1	94.9 ± 33.3*	112.0 ± 27.7^†^	< 0.001
LVOT VTI, cm	21.4 ± 4.1	16.0 ± 4.4*	21.2 ± 3.7	< 0.001
LVOT diameter, mm	21.0 ± 1.5	21.8 ± 1.9	21.2 ± 1.6	0.06
LVOT diameter/BSA	12.9 ± 1.3	13.1 ± 1.6	13.0 ± 1.3	0.55
AVA_echo_, mm^2^	61.3 ± 14.7	66.9 ± 15.1	69.5 ± 13.8^†^	< 0.001
ESVI, mL/m^2^	27.7 ± 16.8	63.4 ± 27.0*	24.5 ± 11.4	< 0.001
EDVI, mL/m^2^	66.8 ± 23.7	96.3 ± 29.3*	64.3 ± 24.5	< 0.001
SAC, mL/m^2^/mmHg	0.8 ± 0.3	0.7 ± 0.3	0.8 ± 0.3	0.28
Zva, mmHg/mL/m^2^	5.3 ± 1.6	5.0 ± 1.9	4.4 ± 1.4^†^	< 0.001
**CT findings**				
Valve morphology				0.04
Tricuspid	204 (46.6)	14 (77.8)	28 (50.9)	
Bicuspid with raphe	106 (24.2)	3 (16.7)	17 (30.9)	
Bicuspid without raphe	128 (29.2)	1 (5.6)	10 (18.2)	
AVC, Agatston unit	3027.2 ± 1872.0	2895.5 ± 1624.5	2363.1 ± 1605.8^†^	0.04
AVC_ratio_^‡^	1.8 ± 1.0	1.6 ± 0.8	1.4 ± 0.8^†^	0.006
LVOT mean diameter	24.8 ± 2.9	27.1 ± 2.7*	24.7 ± 2.6	0.003
AVA_CT_, mm^2^	84.9 ± 23.4	100.8 ± 22.7*	94.2 ± 25.0^†^	0.001
AVA_plani_, mm^2^	87.2 ± 23.2	99.7 ± 25.5*	97.5 ± 27.1^†^	0.003
Aortic annulus				
Circularity, %	81.6 ± 7.6	77.4 ± 6.6	82.3 ± 6.0	0.05
Maximal dimeter, mm	27.5 ± 3.2	30.4 ± 3.5*	27.4 ± 2.8	0.001
Mean diameter, mm	24.9 ± 2.6	26.9 ± 2.5*	25.0 ± 2.6	0.005
Perimeter, mm	79.5 ± 8.4	85.4 ± 7.7*	80.0 ± 8.3	0.02
Area, mm^2^	481.8 ± 102.4	554.8 ± 101.0*	489.5 ± 99.9	0.01
Sinus of Valsalva, mm	36.6 ± 4.5	38.4 ± 4.7	36.8 ± 5.0	0.25
Sinotubular junction, mm	30.9 ± 4.6	31.7 ± 3.3	31.5 ± 5.9	0.58
Ascending aorta tubular portion, mm	40.7 ± 6.3	38.4 ± 4.6	40.4 ± 7.7	0.31
**Normalized to BSA**				
AVA_CT_, mm^2^	51.7 ± 13.6	60.7 ± 15.8*	58.0 ± 15.6^†^	< 0.001
AVA_plani_, mm^2^	52.6 ± 13.5	59.5 ± 17.5*	57.8 ± 15.2^†^	0.005
Aortic annulus				
Maximal dimeter, mm	16.8 ± 2.0	18.2 ± 2.6*	16.9 ± 1.7	0.01
Mean diameter, mm	15.2 ± 1.6	16.1 ± 1.9	15.4 ± 1.6	0.06
Perimeter, mm	48.7 ± 5.2	51.1 ± 5.8	49.2 ± 5.3	0.12
Area, mm^2^	293.4 ± 55.5	331.0 ± 56.6*	299.7 ± 55.9	0.02
Sinus of Valsalva, mm	22.4 ± 2.9	23.0 ± 2.8	22.6 ± 2.9	0.65
Sinotubular junction diameter, mm	18.9 ± 2.9	18.9 ± 2.1	19.3 ± 3.2	0.69
Ascending aorta tubular portion, mm	25.0 ± 4.3	23.0 ± 2.9	24.8 ± 4.6	0.15
Surgical valve size, mm	22.1 ± 2.1	23.0 ± 2.1	22.3 ± 2.0	0.18
**Surgical valve type**				N/A
CE Magna	144	10	18	
ATSAP	82	4	8	
Hancock	82	1	13	
St. Jude Regent	80	3	11	
Others	50	0	5	
**Operator**				0.47
Operator 1	170	5	18	
Operator 2	95	2	12	
Operator 3	81	4	15	
Operator 4	64	4	7	
Operator 5	28	3	3	
MACCE	34 (7.8)	5 (27.8)*	4 (7.3)	0.01
All-cause mortality	57 (13.0)	6 (33.3)	8 (14.5)	0.05
Follow-up duration, d	1517.5 (1188.8–2026.5)	1134.0 (26.0–1682.0)	1455.0 (1112.0–1944.0)	0.007

Values are means ± standard deviations or numbers and percentages in parentheses.

*Significant difference between patients with high-gradient severe aortic stenosis and patients with classic LF-LG AS groups.

^†^Significant difference between high-gradient severe aortic stenosis and paradoxical LF-LG AS groups.

^‡^Value divided by sex-specific thresholds (Male, 2000; Female, 1250).

*AS* aortic stenosis, AV aortic valve, *AVA* aortic valve area, *AVC* aortic valve calcium score, *BNP* B-type natriuretic peptide, CABG coronary artery bypass graft, *EDVI* end-diastolic volume index, *ESVI* end-systolic volume index, *LF-LG* low-flow and low-gradient, *lnBNP* log-transformed B-type natriuretic peptide, *LVEF* left ventricular ejection fraction, *LVMI* left ventricular mass index, *LVOT* left ventricular outflow tract, *MACCE* major adverse cardiac and cerebrovascular event, *N/A* not available, PCI percutaneous coronary artery intervention, *PG* pressure gradient, *SAC* systemic arterial compliance, *VTI* velocity time integral, *Zva* valvulo-arterial impedance.

Among the groups with high-gradient severe AS, classic LF-LG AS, and paradoxical LF-LG AS, the age of patients was not statistically different (P = 0.93) (Table 1). The number of concurrent percutaneous coronary artery intervention or coronary artery bypass graft with AVR was highest in classic LF-LG AS group (50%, P = 0.02). B-type natriuretic peptide (BNP) was highest in classic LF-LG AS (median 944.5 pg/mL, P < 0.001). MACCE was more common in the classic LF-LG AS than in the high-gradient severe AS (27.8 vs. 7.8%, P = 0.01).

### Echocardiography

LVEF, transaortic peak velocity and PG were lower in the classic LF-LG AS group and reflected the characteristics of LF-LG AS (P < 0.001, for all) (Table 1). The end-systolic volume index (ESVI) (63.4 vs. 27.7 mL/m^2^, P < 0.001) and end-diastolic volume index (EDVI) (96.3 vs. 66.8 mL/m^2^, P < 0.001) were significantly larger in classic LF-LG AS, compared to the high-gradient severe AS group. Systemic arterial compliance was not different among the groups (P = 0.28), although valvulo-arterial impedence (Zva) was lower in paradoxical LF-LG AS compared to others (P < 0.001).

**Figure 1:**
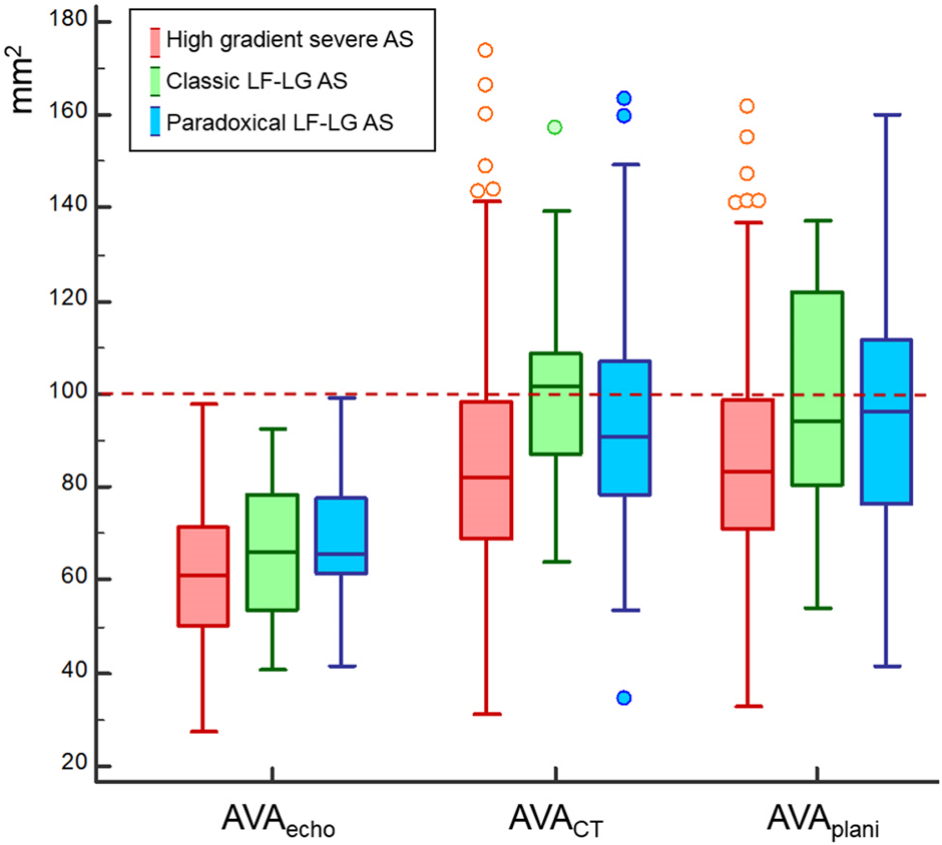
Box plot to demonstrate the distribution of AVA_echo_, AVA_CT_, and AVA_plani_ according to categories of AS. *AS* aortic stenosis, *AVA* aortic valve area, *LF-LG* low-flow and low-gradient.

We found that AVA_CT_ ≥ 1.2 cm^2^ was noted in 9.8% (43/438) of the patients with high-gradient severe AS, 27.8% (5/18) of with classic LF-LG AS, and 14.5% of paradoxical LF-LG AS (Fig. 1). In high-gradient severe AS, patients with AVA_CT_ ≥ 1.2 cm^2^ also showed larger AVA_echo_ (80.4 vs. 59.0 mm^2^, P < 0.001) with higher LVOT velocity time integral (VTI) (22.6 vs. 21.3 cm, P = 0.04) and lower AV VTI (101.0 vs. 127.2 cm, P < 0.001) than those of with AVA_CT_ < 1.2 cm^2^. The LVOT diameter had no significant different between two groups (21.4 vs. 21.0, P = 0.10) (Table 2). In classic LF-LG AS, AVA_echo_ was larger in patients with AVA_CT_ ≥ 1.2 cm^2^ than those with AVA_CT_ < 1.2 cm^2^ (61.1 vs. 81.9 mm^2^, P = 0.005). However, other echocardiography parameters such as LVEF, peak velocity, and PG were not statistically different between subgroups with AVA_CT_ < 1.2 cm^2^ and AVA_CT_ ≥ 1.2 cm^2^ (P > 0.05, for all). In patients with paradoxical LF-LG AS, AVA_echo_ was larger in AVA_CT_ ≥ 1.2 cm^2^ group (68.2 vs. 77.3 mm^2^, P = 0.08), but without statistical significance.

**Table 2:** Subgroups of LF-LG AS according to AVA_CT_.

Characteristic	High-gradient severe AS (n = 438)			Classic LF-LG AS (n = 18)			Paradoxical LF-LG AS (n = 55)		
	AVA_CT_ < 1.2 cm^2^	AVA_CT_ ≥ 1.2 cm^2^	P-value	AVA_CT_ < 1.2 cm^2^	AVA_CT_ ≥ 1.2 cm^2^	P-value	AVA_CT_ < 1.2 cm^2^	AVA_CT_ ≥ 1.2 cm^2^	P-value
No. of patients (%)	395 (90.2)	43 (9.8)		13 (72.2)	5 (27.8)		47 (85.5)	8 (14.5)	
Age, years	66.8 ± 8.9	67.0 ± 8.4	0.92	66.1 ± 5.8	68.6 ± 7.7	0.46	67.3 ± 9.2	67.3 ± 10.9	0.99
Male	201 (50.9)	35 (81.4)	< 0.001	9 (69.2)	4 (80.0)	1.00	25 (53.2)	6 (75.0)	0.72
BSA, m^2^	1.6 ± 0.2	1.7 ± 0.2	< 0.001	1.7 ± 0.2	1.6 ± 0.2	0.12	1.6 ± 0.2	1.7 ± 0.1	0.34
Hypertension	208 (52.7)	23 (53.5)	1.00	7 (53.8)	5 (100.0)	0.11	26 (55.3)	4 (50.0)	1.00
Atrial fibrillation	59 (14.9)	6 (14.0)	1.00	3 (23.1)	1 (20.0)	1.00	2 (4.3)	2 (25.0)	0.10
PCI or CABG	88 (22.3)	6 (14.0)	0.29	6 (46.2)	3 (60.0)	1.00	14 (25.5)	0 (0.0)	0.10
Rheumatic valvular disease	37 (9.4)	1 (2.3)	0.12	2 (15.4)	0 (0.0)	1.00	6 (12.8)	2 (25.0)	0.33
B-type natriuretic peptide, pg/mL	106.0 (49.0 –282.5)	51.0 (31.0 –190.5)	0.05	1118.0 (304.8–3066.0)	738.5 (239.3–3227.5)	0.80	59.0 (32.5–180.5)	200.5 (59.0–305.3)	0.38
lnBNP	4.7 (3.9–5.6)	3.9 (3.4–5.2)	0.05	7.0 (5.7–8.0)	6.6 (5.0–7.9)	0.60	4.1 (3.5–5.2)	5.2 (4.1–5.7)	0.12
Blood urea nitrogen, mg/dL	17.7 ± 7.2	20.4 ± 10.2	0.10	25.2 ± 16.7	18.6 ± 4.7	0.22	18.8 ± 8.5	18.0 ± 4.7	0.81
Creatinine, mg/dL	0.9 ± 0.6	1.4 ± 1.9	0.09	2.1 ± 2.3	1.9 ± 1.3	0.76	1.0 ± 0.4	0.9 ± 0.1	0.46
**Echocardiography**									
LVEF, %	60.3 ± 10.1	60.8 ± 8.9	0.72	36.9 ± 11.2	33.8 ± 8.0	0.59	62.8 ± 5.4	61.5 ± 4.6	0.53
Peak velocity, m/s	5.2 ± 0.7	4.9 ± 0.6	0.006	3.6 ± 0.4	3.4 ± 0.7	0.37	3.5 ± 0.5	3.6 ± 0.6	0.67
Peak PG, mmHg	109.8 ± 31.0	96.9 ± 22.4	0.001	53.5 ± 11.7	48.0 ± 17.0	0.44	49.5 ± 16.0	53.4 ± 16.9	0.53
Mean PG, mmHg	67.3 ± 19.7	58.5 ± 14.7	0.001	30.8 ± 6.8	27.2 ± 10.6	0.41	27.7 ± 9.4	29.8 ± 10.2	0.59
LVMI, g/m^2^	135.4 ± 35.8	134.8 ± 36.5	0.91	151.5 ± 33.5	144.7 ± 26.3	0.69	121.5 ± 32.7	144.6 ± 50.6	0.10
AV VTI, cm	127.2 ± 25.7	101.0 ± 16.5	< 0.001	101.3 ± 34.2	78.2 ± 26.9	0.20	114.2 ± 28.2	98.9 ± 21.3	0.15
LVOT VTI, cm	21.3 ± 4.1	22.6 ± 3.6	0.04	16.0 ± 4.8	16.2 ± 3.8	0.92	21.4 ± 3.6	19.7 ± 3.7	0.24
LVOT diameter, mm	21.0 ± 1.5	21.4 ± 1.5	0.10	22.1 ± 2.2	21.2 ± 0.2	0.22	21.0 ± 1.4	22.2 ± 2.2	0.18
LVOT diameter/BSA	12.9 ± 1.3	12.3 ± 1.0	< 0.001	12.9 ± 1.6	13.6 ± 1.4	0.40	13.0 ± 1.4	13.2 ± 1.2	0.75
AVA_echo_, mm^2^	59.0 ± 13.6	80.4 ± 8.8	< 0.001	61.1 ± 12.8	81.9 ± 9.1	0.005	68.2 ± 13.6	77.3 ± 12.7	0.08
ESVI	27.7 ± 16.9	28.0 ± 16.4	0.92	63.0 ± 30.0	64.4 ± 20.0	0.92	23.4 ± 10.6	30.7 ± 14.5	0.10
EDVI	66.6 ± 23.4	68.7 ± 26.7	0.57	96.4 ± 32.6	95.9 ± 21.3	0.98	62.0 ± 22.6	78.4 ± 31.5	0.08
SAC, mL/m^2^/mmHg	0.8 ± 0.3	0.8 ± 0.3	0.11	0.7 ± 0.3	0.6 ± 0.1	0.52	0.7 ± 0.3	1.0 ± 0.5	0.21
Zva, mmHg/mL/m^2^	5.4 ± 1.6	5.2 ± 1.7	0.51	4.7 ± 1.7	5.8 ± 2.2	0.82	4.5 ± 1.4	3.8 ± 1.5	0.21
**CT findings**									
Valve morphology			0.04			0.28			0.14
Tricuspid	177 (44.8)	27 (62.8)		9 (69.2)	5 (100.0)		26 (55.3)	2 (25.0)	
Bicuspid	218 (55.2)	16 (37.2)		4 (30.8)	0 (0.0)		21 (44.7)	6 (75.0)	
AVC, Agatston unit	2834.2 (1605.2–4178.4)	2426.7 (1616.1–3808.9)	0.57	3912.2 (2247.0–4496.1)	1360.1 (960.1–2108.6)	0.002	2002.4 (1192.2–2841.6)	2584.3 (1306.5–5172.0)	0.12
AVA_CT_	80.9 ± 18.8	136.6 ± 29.6	< 0.001	92.4 ± 15.5	122.7 ± 25.2	0.007	90.2 ± 22.4	117.7 ± 27.9	0.003
AVA_plani_, mm^2^	85.2 ± 22.1	105.0 ± 26.1	< 0.001	88.2 ± 20.0	129.9 ± 5.4	< 0.001	90.1 ± 21.2	140.9 ± 13.8	< 0.001
LVOT area	346.8 ± 50.0	359.6 ± 51.3	0.11	587.5 ± 129.7	572.3 ± 79.9	0.81	468.9 ± 94.5	581.3 ± 94.9	0.003
Aortic annulus									
Maximum diameter, mm	27.1 ± 3.0	30.6 ± 3.2	< 0.001	30.6 ± 3.9	30.0 ± 2.1	0.77	27.1 ± 2.6	29.0 ± 3.6	0.05
Circularity, %	80.0 ± 1.0	80.0 ± 1.0	0.22	77.6 ± 7.5	76.9 ± 4.4	0.84	82.0 ± 5.7	83.8 ± 7.6	0.18
Mean diameter, mm	24.6 ± 2.5	27.5 ± 2.6	< 0.001	27.1 ± 2.9	26.5 ± 1.4	0.70	24.7 ± 2.3	27.0 ± 3.0	0.01
Perimeter, mm	78.7 ± 8.0	87.3 ± 8.6	< 0.001	86.0 ± 8.9	83.8 ± 3.7	0.46	78.7 ± 7.4	87.3 ± 10.0	0.01
Area, mm^2^	471.4 ± 95.0	576.8 ± 119.4	< 0.001	563.3 ± 116.1	532.8 ± 45.9	0.44	475.2 ± 91.3	572.9 ± 113.2	0.009
Sinus of Valsalva, mm	36.2 ± 4.5	39.4 ± 3.8	< 0.001	39.3 ± 4.9	36.1 ± 3.4	0.20	35.9 ± 4.6	42.0 ± 4.2	0.001
Sinotubular junction, mm	30.7 ± 4.6	32.8 ± 4.4	0.004	32.1 ± 3.5	30.7 ± 2.8	0.44	30.6 ± 5.1	36.7 ± 7.7	0.006
Ascending aorta, mm	40.7 ± 6.3	41.0 ± 5.8	0.74	39.5 ± 4.4	35.5 ± 4.2	0.10	39.6 ± 7.1	45.1 ± 9.8	0.06
**Normalized to BSA**									
AVA_CT_	51.8 ± 13.0	59.7 ± 15.7	< 0.001	53.9 ± 9.4	78.3 ± 16.1	0.001	55.9 ± 14.3	70.3 ± 17.9	0.01
AVA_plani_, mm^2^	52.4 ± 13.5	60.9 ± 16.4	< 0.001	51.5 ± 12.3	82.7 ± 6.0	< 0.001	55.7 ± 12.7	84.1 ± 10.4	< 0.001
LVOT area, mm^2^	294.1 ± 61.4	322.3 ± 62.4	0.005	341.2 ± 69.4	365.5 ± 54.6	0.50	289.3 ± 55.3	345.5 ± 54.4	0.01
Aortic annulus									
Maximal dimeter, mm	16.7 ± 1.9	17.7 ± 2.0	0.002	17.9 ± 2.8	19.2 ± 2.0	0.35	16.8 ± 1.7	17.4 ± 1.6	0.37
Mean diameter, mm	15.2 ± 1.6	15.9 ± 1.6	0.006	15.8 ± 2.0	17.0 ± 1.7	0.27	15.3 ± 1.6	16.1 ± 1.6	0.20
Perimeter, mm	48.5 ± 5.2	50.4 ± 5.1	0.02	50.2 ± 6.0	53.6 ± 5.0	0.28	48.7 ± 4.9	52.0 ± 6.8	0.10
Area, mm^2^	289.3 ± 53.4	331.3 ± 60.0	< 0.001	327.6 ± 65.3	339.7 ± 26.4	0.70	292.7 ± 50.4	341.4 ± 71.4	0.02
Sinus of Valsalva	36.2 ± 4.5	39.4 ± 3.8	< 0.001	22.9 ± 3.0	23.0 ± 2.4	0.95	22.1 ± 2.6	25.1 ± 3.5	0.007
Sinotubular junction diameter	30.7 ± 4.6	32.8 ± 4.4	0.004	18.7 ± 1.9	19.6 ± 2.5	0.40	18.9 ± 2.5	21.9 ± 5.3	0.01
Ascending aorta tubular portion	40.7 ± 6.3	41.0 ± 5.8	0.74	23.1 ± 3.0	22.6 ± 2.9	0.78	24.5 ± 4.3	26.9 ± 5.7	0.18
Surgical valve size, mm	21.9 ± 2.1	23.5 ± 1.8	< 0.001	23.2 ± 2.3	22.6 ± 1.7	0.63	22.1 ± 2.1	23.3 ± 1.7	0.14
MACCE (cardiovascular death)	30 (7.6)	4 (9.3)	0.92	2 (15.4)	3 (60.0)	0.10	4 (8.5)	0 (0.0)	1.00
All-cause mortality	52 (13.2)	5 (11.6)	0.96	4 (30.8)	2 (40.0)	1.00	7 (14.9)	1 (12.5)	1.00

AS, aortic stenosis; AVA, aortic valve area; AVA_CT_, AVA measured on CT; AVC, aortic valve calcium score; BSA, body surface area; CABG, coronary artery bypass graft; EDVI, end-diastolic volume index; ESVI, end-systolic volume index; LF-LG, low-flow and low-gradient; lnBNP, log-transformed B-type natriuretic peptide; LVEF, left ventricular ejection fraction; LVMI, left ventricular mass index; LVOT, left ventricular outflow tract; MACCE, major adverse cardiac and cerebrovascular event; PCI, percutaneous coronary artery intervention; VTI, velocity time integral.

### Comparison of AVA measured by echocardiography and CT

**Figure 2:**
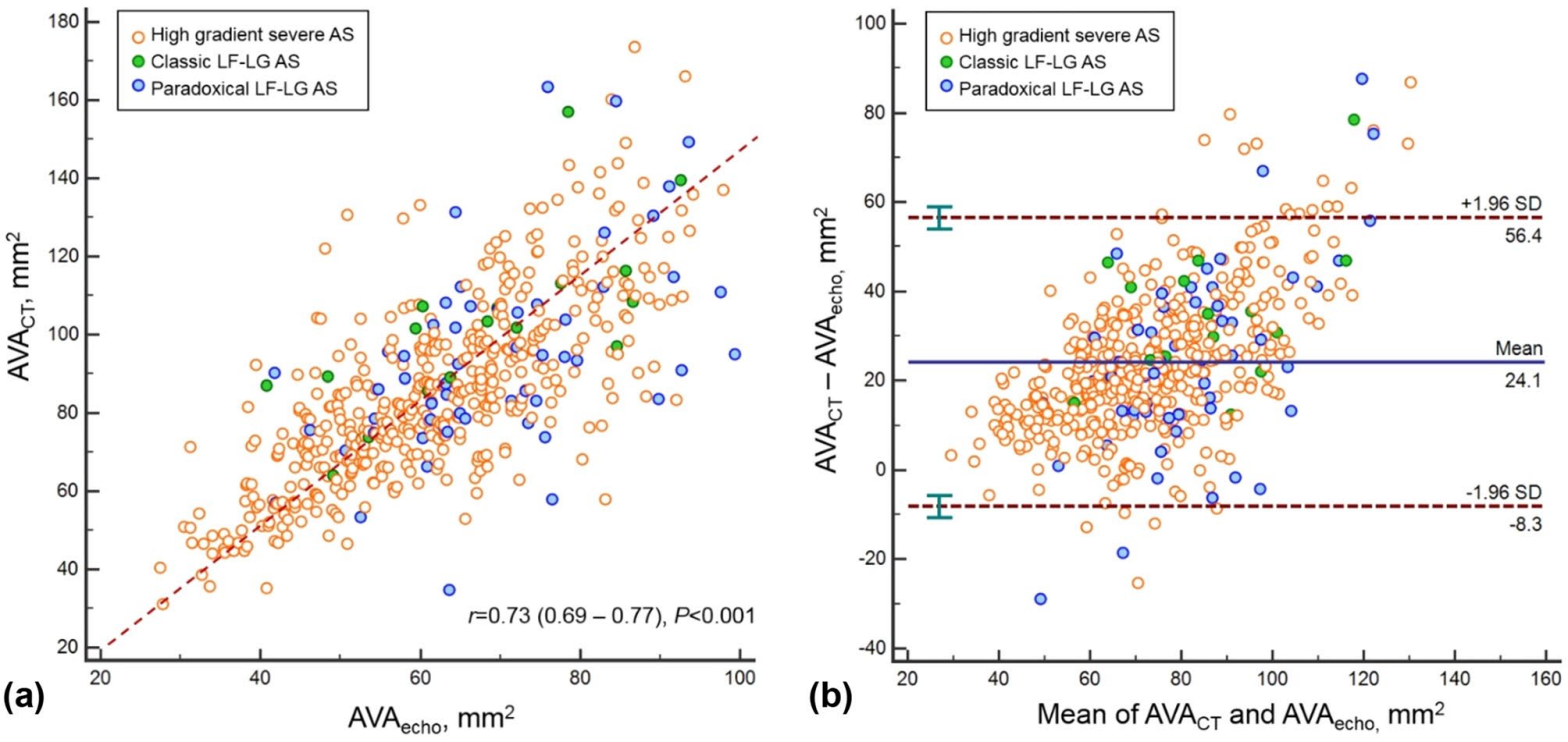
(**a**) Pearson correlation analysis result and (**b**) Bland–Altman plot to comparison of AVA_CT_ and AVA_echo_. *AS* aortic stenosis, *AVA* aortic valve area, *LF-LG* low-flow and low-gradient.

Interobserver agreements for aortic root measurement on CT are high with the range of intra-class correlation coefficient (ICC) from 89.2 to 97.0 (Supplementary Table 2). The Pearson correlation coefficient for AVA_echo_ and AVA_CT_ was good (r = 0.73, P < 0.001). AVA_CT_ is larger than AVA_echo_ and the mean difference between AVA_echo_ and AVA_CT_ was 24.1 mm^2^ (95% confidence interval [CI], − 8.3 to 56.4 mm^2^, P < 0.001) (Fig. 2A,B). Comparison of AVA_echo_ and AVA_plani_ is presented in Supplementary Fig. 2.

### CT findings according to AS subtypes

**Figure 3:**
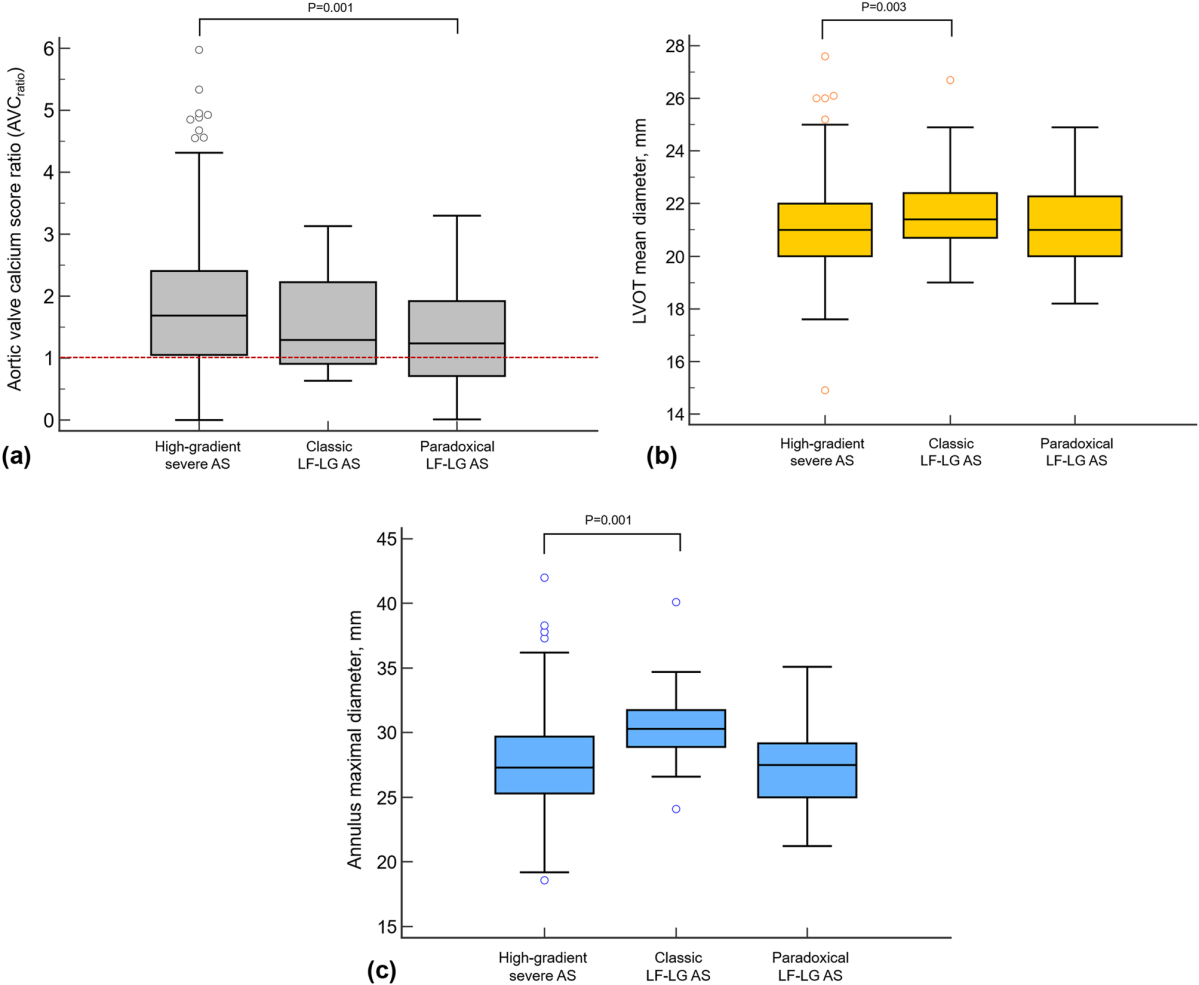
Box plot to demonstrate the distribution of AVC_ratio_, LVOT mean diameter, and maximal diameter of aortic annulus according to subtypes. (**a**) AVC_ratio_ was calculated by dividing AVC with sex-specific thresholds (Male, 2000; Female, 1250). The score above the red-dotted line represents AVC above the sex-specific threshold, and consequently, severe aortic stenosis. The score below the red-dotted line represents the AVC below the sex-specific threshold and nonsevere calcification. The mean of AVC_ratio_ was significantly lower in paradoxical LF-LG AS than that of high gradient severe AS (P = 0.001). In addition, the proportion of nonsevere calcification was most frequent in paradoxical LF-LG AS patients. Both (**b**) LVOT mean diameter and (**c**) maximal diameter of aortic annulus were lowest in LF-LG AS patients among the three subtypes and significantly larger than those of high-gradient severe AS patients. *AS* aortic stenosis, *AVC* aortic valve calcium score, *LF-LG* low-flow and low-gradient, *LVOT* left ventricular outflow tract.

AVC was highest in patients with high-gradient severe AS, and statistically lower in paradoxical LF-LG AS (P = 0.04). When adjusted to sex-specific threshold, AVC_ratio_ was lowest in patients with paradoxical LF-LG AS and lower than that of high-gradient severe AS (1.8 vs. 1.4, P = 0.006, Fig. 3A). LVOT mean dimeter measured on CT was largest in LF-LG AS group and larger than that in high-gradient severe AS (24.8 vs. 27.1 mm, P = 0.003, Fig. 3B). The maximal diameter of aortic annulus largest in classic LF-LG AS group and larger than that of high-gradient severe AS (27.5 vs. 30.4 mm, P = 0.001, Fig. 3C). The mean AVA_CT_ was larger in the classic LF-LG AS group, compared to the high-gradient severe AS group (100.8 vs. 84.9 mm^2^, P = 0.001).

With cut-off value of AVA_CT_ < 1.2 cm^2^, 455 of 511 (89.0%) patients were categorized as severe AS. However, 56 patients were re-classified as moderate AS (43 [9.8%] high-gradient severe AS, 5 [27.8%] classic LF-LG AS, and 8 [14.5%] paradoxical LF-LG AS). In high-gradient severe AS group, the re-classified moderate AS patients had larger normalized measurements of annulus than those in concordant severe AS (P < 0.05, for all). Normalized LVOT area was also larger in re-classified moderate AS (322.3 vs. 294.1 mm^2^, P = 0.005) (Table 2). In classic LF-LG AS group, severe AS patients who showed concordance between echocardiography and CT (AVA_CT_ < 1.2 cm^2^) had higher mean AVC (3912.2 vs. 1360.1, P = 0.002) and smaller AVA_plani_ (88.2 vs. 129.9 mm^2^, P < 0.001) than those in re-classified moderate AS patients (AVA_CT_ ≥ 1.2 cm^2^). The normalized annulus sizes and aortic root diameters on CT were not statistically different between concordant severe AS and re-classified moderate AS groups (P < 0.05, for all). In paradoxical LF-LG AS group, LVOT area normalized to body surface area (BSA) (289.3 vs. 345.5 mm^2^, P = 0.01), normalized sizes of aortic annulus area (292.7 vs. 341.4 mm^2^, P = 0.02), sinus of Valsalva (22.1 vs. 25.2 mm, P = 0.003) and ST junction (30.6 vs. 36.7 mm, P = 0.006) were larger in concordant severe AS patients, compared to those measured in re-classified moderate AS patients.

### Outcome analysis

Of 511 patients, the all-cause mortality was 13.9% (n = 71) and MACCE occurred 8.4% (n = 43). MACCE were composed of major arrhythmia requiring treatment (n = 6), nonfatal cerebrovascular accident (n = 10), nonfatal myocardial infarction (n = 4), heart failure (n = 4), reoperation (n = 1), and cardiovascular death (n = 18). To identify clinical and radiological factors that affect MACCE, cox-proportional hazard regression analysis was performed (Table 3). In univariate analysis, older age, high BNP, high blood urea nitrogen and creatinine, presence of preoperative atrial fibrillation (AF), tricuspid AV, classic LF-LG AS, small AV VTI and LVOT VTI, and small aortic annulus were factors significantly associated with MACCE (P < 0.05, for all).

**Table 3:** Cox proportional hazard regression model for prediction of MACCE.

Parameter	Univariate		Multivariable	
	HR (95% CI)	P-value	HR (95% CI)	P-value
Age, years	1.06 (1.02–1.10)	0.005	1.04 (1.00–1.09)	0.049
BSA, m^2^	0.49 (0.08–3.15)	0.45		
B-type natriuretic peptide, 10 pg/mL	1.01 (1.003–1.01)	< 0.001	1.005 (1.003–1.01)	< 0.001
lnBNP	1.42 (1.13–1.77)	0.002		
Atrial fibrillation, (%)	3.22 (1.70–6.11)	< 0.001	2.75 (1.40–5.40)	0.003
LVEF, %	0.98 (0.96–1.01)	0.20		
Peak velocity, m/s	0.80 (0.57–1.12)	0.19		
Mean PG, mmHg	0.99 (0.98–1.01)	0.21		
LVMI, g/m^2^	0.99 (0.99–1.00)	0.18		
ESVI, mL/m^2^	1.01 (0.99–1.02)	0.55		
EDVI, mL/m^2^	1.00 (0.99–1.01)	0.90		
SAC, mL/m^2^/mmHg	0.57 (0.21–1.53)	0.27		
Zva, mmHg/mL/m^2^	1.00 (0.83–1.20)	0.98		
Bicuspid aortic valve	0.40 (0.21–0.77)	0.006		
Classic LF-LG AS	5.04 (1.98–12.84)	0.001	5.53 (1.74–17.56)	0.004
AVA_echo_, m^2^	1.01 (0.99–1.03)	0.60		
AV VTI, cm	0.98 (0.96–0.99)	< 0.001		
LVOT VTI, cm	0.92 (0.86–0.99)	0.02		
lnAVC	0.89 (0.71–1.09)	0.26		
Normalized AVA_plani_, mm^2^	1.00 (0.98–1.03)	0.76		
Normalized AVA_CT_, mm^2^	1.02 (1.00–1.04)	0.50		
Annulus circularity, %	0.34 (0.01–18.69)	0.59		
Aortic annulus area, cm^2^	0.73 (0.53–1.01)	0.06	0.57 (0.40–0.81)	0.002
Surgical valve size, mm	0.89 (0.77–1.04)	0.13		
**Surgical valve type**				
CE Magna	1	0.63		
ATSAP	0.55 (0.20–1.48)	0.24		
Hancock	1.09 (0.50–2.38)	0.83		
St. Jude Regent	0.63 (0.25–1.59)	0.32		
Others	0.85 (0.31–2.31)	0.75		
**Operator**				
Operator 1	1	0.33		
Operator 2	1.63 (0.78–3.38)	0.19		
Operator 3	0.37 (0.05–2. 76)	0.33		
Operator 4	0.94 (0.37–2.41)	0.90		
Operator 5	0.73 (0.30–1.78)	0.49		

*AS* aortic stenosis, *AVA* aortic valve area, *AVC* aortic valve calcium score, CI confidence interval, *EDVI* end-diastolic volume index, *ESVI* end-systolic volume index, *HR* hazard ratio, *LF-LG* low-flow and low-gradient, *lnBNP* log-transformed B-type natriuretic peptide, *LVEF* left ventricular ejection fraction, *LVMI* left ventricular mass index, *LVOT* left ventricular outflow tract, *MACCE* major adverse cardiac and cerebrovascular event, *PG* pressure gradient, *SAC* systemic arterial compliance, *VTI* velocity time integral, *Zva* valvulo-arterial impedance.

On multivariable analysis, old age (hazard ratio^10^, 1.04, 95% CI, 1.00–1.09; P = 0.049), high BNP (HR, 1.005; 95% CI, 1.003–1.01 P < 0.001), AF (HR, 2.75; 95% CI, 1.40–5.40; P = 0.003), classic LF LG AS (HR, 5.53; 95% CI, 1.74–17.56; P = 0.004), and small aortic annulus area (cm^2^), [HR, 0.57; 95% CI, 0.40–0.81; P = 0.002]) were factors significantly associated with MACCE (Table 3). Normalized aortic annulus area (cm^2^) (HR, 0.40; 95% CI, 0.22–0.74; P = 0.004) was also a significantly associated factor when the parameter was substitute instead of aortic annulus area in multivariable analysis. When the normalized aortic sinus of Valsalva diameter instead of the aortic annulus size was substituted in the calculation, the weight of the other factors remained almost unchanged, while the normalized aortic sinus of Valsalva diameter (cm) (HR, 0.30; 95% CI, 0.09–0.98; P = 0.04) was also identified as a significant factor.

**Figure 4:**
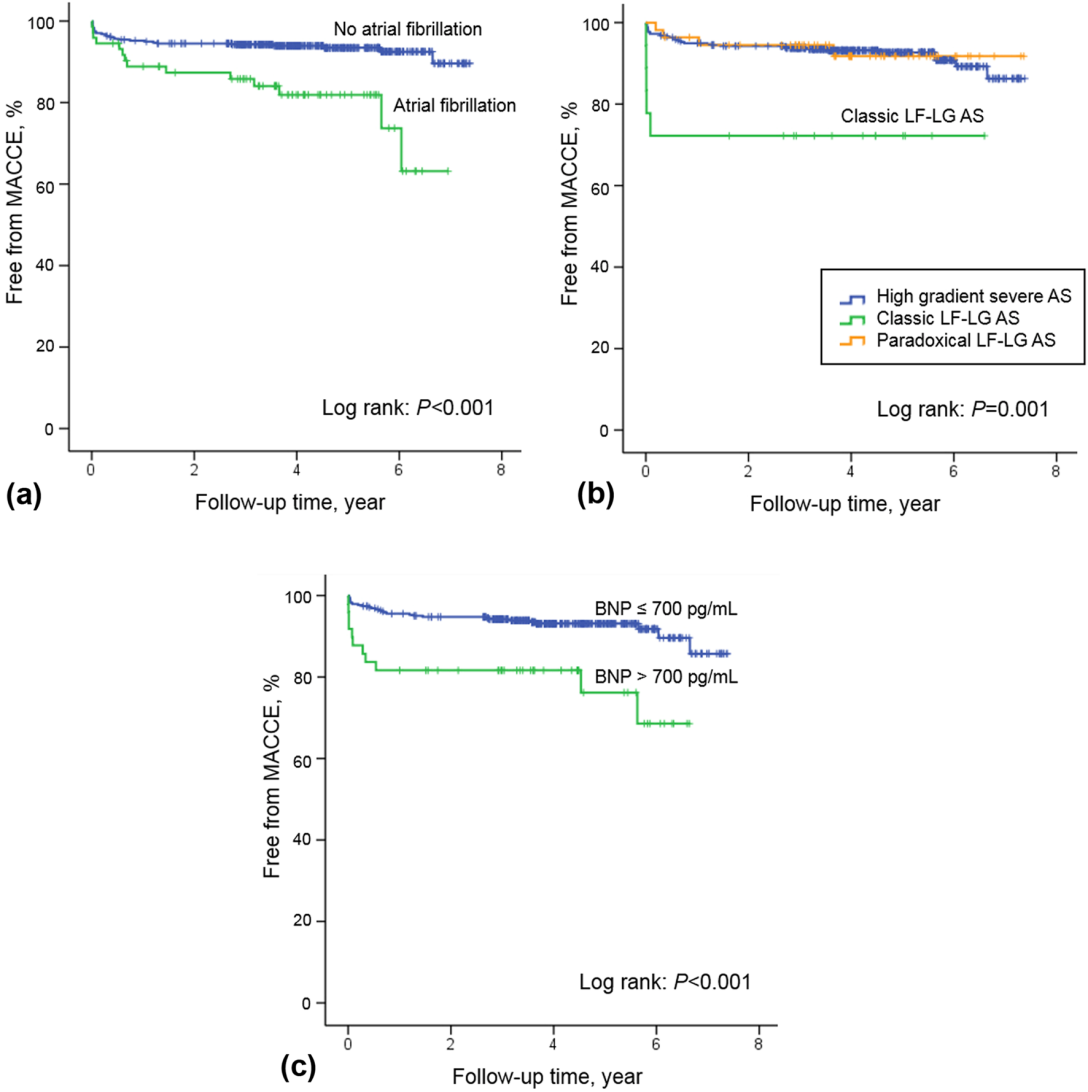
Survival according to (**a**) B-type natriuretic peptide, (**b**) presence of atrial fibrillation, and (**c**) categories of aortic stenosis. *AS* aortic stenosis, *LF-LG* low-flow and low-gradient.

Kaplan–Meier curves indicated significant mortality in the high BNP group (BNP > 700 pg/mL) compared to the low BNP group (P = 0.001) (Fig. 4A). Furthermore, preoperative AF was also associated with significant mortality (Fig. 4B) (P < 0.001), and the outcome of classic LF-LG AS was worse in the cumulative survival curve (Fig. 4C) (P = 0.001).

## Discussion

Herein, we described preoperative CT characteristics of different subtypes of AS, compared AVA_CT_ with AVA_echo_, and identified prognostic factors after AVR. AVA_echo_ and AVA_CT_ showed high concordance rate (89.0%) to classify severe AS, except 56 patients who were re-classified as moderate AS (43 [9.8%] high-gradient severe AS, 5 [27.8%] classic LF-LG AS, and 8 [14.5%] paradoxical LF-LG AS) by AVA_CT_. The AVA_CT_ and aortic annulus were larger in classic LF-LG AS compared to those in high-gradient severe AS. High BNP, preoperative AF, classic LF-LG AS, and smaller aortic root were associated with MACCE after AVR.

**Figure 5:**
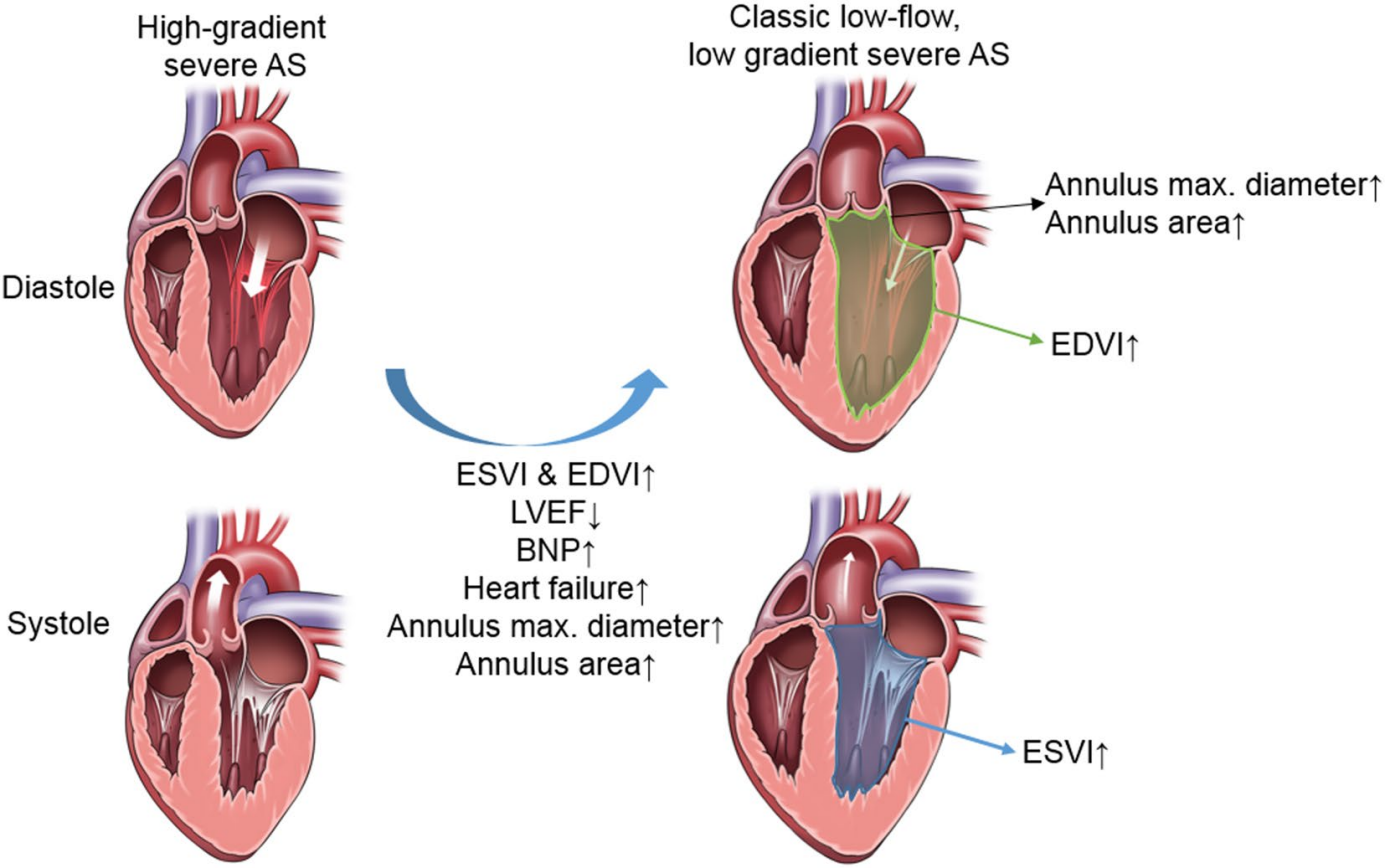
Characteristics of classic LF-LG AS. Classic LF-LG AS patients demonstrated higher ESVI and EDVI, lower LVEF, larger aortic annulus diameter and area compared to high-gradient severe AS. The drawings were prepared using Photoshop 2019 (version 20). *AS* aortic stenosis, *BNP* B-type natriuretic peptide, *EDVI* end-diastolic volume index, *ESVI* end-systolic volume index, *LVEF* left ventricular ejection fraction.

Among the three different subtypes of severe AS, classic LF-LG AS patients demonstrated higher ESVI and EDVI, lower LVEF, larger AVA_echo_ and AVA_CT_, and larger aortic annulus compared to high-gradient severe AS. In a previous study, patients with severe AS had significantly larger aortic annulus and sinotubular (ST) junction diameters compared with those measured in control groups^14^. Compensatory increment of ESVI and EDVI and subsequent LV dilatation may lead to aortic root remodelling, the dilatation of aortic annulus (Fig. 5). They also had larger LVOT mean diameter and aortic annulus maximal diameter on CT compared to high-gradient severe AS, which could be explained by dilated LV in classic LF-LG AS. Importantly, ESVI, EDVI, and BNP were significantly higher in LF-LG AS than those of high-gradient severe AS. This suggest the adverse remodelling may occur in LF-LG AS and is line with previous description on LF-LG, which shows dilated LV with LV dysfunction^2^. Classic LF-LG AS may be a compensation failure of high-gradient severe AS whereas paradoxical LF-LG AS presented preserved ESVI, EDVI, and LVEF, although AVA_echo_ and AVA_CT_ were larger than in high-gradient severe AS.

In terms of AVC, mean of the AVC_ratio_ was lowest in paradoxical LF-LG AS followed by classic LF-LG AS. That means nonsevere calcification is more frequent in LF-LG AS patients than those of high-gradient severe AS and implies that other factors (e.g., different hemodynamics in AS subtypes) rather than calcification burden could affect the decreased AVA in LF-LG. Although the current diagnostic standard for AS grading and classification is echocardiography, these CT-derived parameters could have a supplementary role in classification of severe AS, especially with poor sonic window or high interobserver variability of echocardiography. We hope the findings based on cardiac CT could provide beginning on further study for prognostic implication of CT-derived parameters, which is less flow-dependent. Further study with large portion of LF-LG AS might reveal different outcomes with AS reclassified by AVA_CT_.

In this study, we used cut-off value of AVA_CT_ < 1.2 cm^2^ as this value was suggested for severe AS in a previous study^12^. In high-gradient severe AS group, approximately 10% (43/438) of patients were re-classified to moderate AS. Different from echocardiography in which the LVOT had no significant difference between concordant and discordant groups, CT revealed larger normalized LVOT area in re-classified moderate AS patients, which probably contribute to discordance. In classic LF-LG AS group, approximately one third of the patients were re-classified to moderate AS. There was no significantly difference of LVOT, of which either measured by echocardiography or CT, in between concordant and discordant groups. Instead, AVC was lower in the AVA_CT_ ≥ 1.2 cm^2^ compared to that of AVA_CT_ < 1.2 cm^2^ group, and in this group, moderate AS patients might be misclassified as severe AS and vice versa. This can also be applied to paradoxical LF-LG patients, despite 14.5% of these patients presenting AVA_CT_ ≥ 1.2 cm^2^. Although we could not derive the role of AVA_CT_ in diagnosing LF-LG AS patients, we consider that further studies with large population of LF-LG AS might reveal different prognosis or post-surgical outcome in patients who reclassified to moderate AS based on AVA_CT_.

The outcome of AS after AVR was associated with preoperative high BNP levels, AF, classic LF-LG AS, and small aortic root. The plasma BNP level was associated with LV dysfunction in AS, and was a well-known predictor of poor outcome in patients with AS overall and after AVR^15–17^. AF is also a dominant predictor in both asymptomatic and symptomatic patients with moderate to severe AS, and after AVR^18–20^. Classic LF-LG AS was associated with worse outcomes after AVR compared those observed in high-gradient AS patients, although LF-LG AS patients have displayed survival benefits with AVR^21^. Finally, small aortic root measured on CT was an independent prognostic factor. This finding should be interpreted cautiously. When AS severity progresses, the increased LV cavity volume may increase the size of the aortic annulus and sinus of Valsalva. However, a small aortic root has also been associated with increased ischemic cardiovascular events and mortality in patients with AS^22^, possibly reflecting impaired root remodelling process and atherosclerotic changes.

Our study has several limitations. Because this is a retrospective study using a patient cohort that underwent AVR, patients not indicated for surgery due to poor general conditions or comorbidities or who declined operation were not included. The selection bias may affect the outcome assessment, and AVR itself was not used as an outcome parameter. Instead, we used MACCE after AVR. Therefore, the outcomes of this study may not directly infer the outcomes of AS population managed with diverse treatment options. Further studies with AS managed by conservative treatment, surgical AVR, and transcatheter AVR could be of value to evaluate overall outcomes of AS patients. Second, we were not able to consider the reverse dynamism of LVOT which could affect the discrepancy between AVA_echo_ and AVA_CT_. Dynamic changes of the diameter of LVOT can result variability of AVA_echo_, but unfortunately it was not routinely evaluated in our institution and the LVOT diameter measured on mid-systolic phase was used for AVA calculation. Third, we showed the CT characteristics of LF-LG AS: AVA_CT_ and aortic annulus were larger in classic LF-LG AS compared to those in high-gradient severe AS. This finding may be explained by the aortic root remodelling which is associated with the dilated LV. However, because of the small number of LF-LG AS patients, we could not generalize the CT findings of LF-LG AS. Further study with larger number of LF-LG AS would be of value. Finally, although classic LF-LG patients showed higher all-cause mortality and a large aortic annulus, a small aortic root was one of the factors associated with MACCE. Both decreased LV function in classic LF-LG AS and impaired aortic root remodelling may contribute to the outcome, respectively, but further studies are necessary to provide more evidence.

In conclusion, AVA_echo_ and AVA_CT_ showed high concordance rate (89.0%) to classify severe AS, however, 56 patients who were re-classified as moderate AS by AVA_CT_. AVC and aortic root size on CT were different among the AS subtypes, high-gradient severe AS, classic LF-LG AS and paradoxical LF-LG AS. Old age, high BNP, AF, classic LF-LG AS and small aortic root on CT were associated with MACCE after AVR. These findings suggest the potential role of cardiac CT in classification and outcome assessment of severe AS.

## Methods

### Patients

This retrospective study was approved by the institutional review board committee of the Asan Medical Center, University of Ulsan College of Medicine (approval number: 2018-0233) and informed consent was waived by the institutional review board due to the retrospective nature of observational study. This study was performed in accordance with the Helsinki Declaration. Between June 2011 and Mar 2016, 781 patients underwent surgical AVR. The use of CT was determined mainly by clinician’s decision, but in our hospital, cardiac CT examination is generally performed in most of the patients who have performed planned surgical AVR for evaluation of AV and root morphology based on the guidelines for the appropriate use of cardiac CT^23–26^. After excluding patients with moderate AS (n = 24), moderate degree of concomitant aortic regurgitation or other valvular heart disease (n = 177), patients not subjected to preoperative cardiac CT (n = 47) or CT without multiphase data (n = 21), and a patient with quadricuspid AV (n = 1), 511 patients were finally included. High-gradient severe AS was defined as AVA_echo_ < 1 cm^2^ and a mean trans-valvular gradient ≥ 40 mmHg with LVEF < 50%. Classic LF-LG severe AS was defined as AVA_echo_ < 1 cm^2^, but with a low-gradient (< 40 mmHg). Low-gradient severe AS with preserved LVEF was defined as paradoxical LF-LG AS. We classified patients with AS into three groups: (1) high-gradient severe; (2) classic LF-LG; and (3) paradoxical LF-LG AS. Clinical findings including age, BSA, hypertension, AF, BNP, echocardiography parameters, and cardiac CT data were collected. Postoperative echocardiography findings and reported clinical outcomes were comprehensively reviewed. Clinical outcomes included all-cause mortality and MACCE (major arrhythmias requiring treatment, composite of cardiac death, cerebrovascular accident or stroke, coronary artery revascularization or myocardial infarction, and redo-AVR) were evaluated. Major arrhythmias included sick sinus syndrome, ventricular fibrillation, and AF/flutter.

### Echocardiography

Preoperatively, all patients underwent transthoracic echocardiography using commercially available ultrasound machines with 3–5 MHz real-time transducers (iE33, EPIC; Philips Medical Systems, Andover, MA; Vivid 7, E9, General Electric Healthcare, Waukesha, WI, USA). Comprehensive two-dimensional and Doppler images were obtained by expert cardiologists according to American Society of Echocardiography recommendations^27^. End-systolic volume, end-diastolic volume, and LVEF were obtained with the biplane Simpson method. The maximal aortic jet velocity was recorded with the apical, right parasternal, or suprasternal window that yielded the highest-velocity signal. The maximal and mean PG across the AV were estimated using a modified Bernoulli equation, and the AVA was calculated from the continuity equation. LV mass and LV mass indexed to BSA calculated by LV cavity dimension and LV wall thickness at end-diastole. SAC was calculated as the ratio of SV index (SVI)/pulse pressure^28^, and valvulo-arterial impedance (Zva), which is a parameter for global LV load, was defined as (systolic blood pressure + mean net aortic gradient)/SVI^29^.

### Cardiac CT protocol and image analysis

Preoperative cardiac CT was performed using a second-generation dual-source CT scanner (Somatom Definition Flash; Siemens Medical Solutions, Forchheim, Germany). Detailed CT protocol is described in Supplementary File 1. Post-processing was conducted using an external workstation (AquariusNet; TeraRecon, Foster City, CA, USA) using multiphase CT data sets reconstructed by a 10% R–R interval. CT analysis methods are described in Supplementary Fig. 1. CT characteristics such as AV morphology (tricuspid, bicuspid with raphe, and bicuspid without raphe), AVA_CT_, AVA obtained by planimetry (AVA_plani_), aortic annulus diameter, perimeter, and area, circularity (minimum annulus diameter/maximum annulus diameter × 100), and diameters of sinus of Valsalva, ST junction, and ascending aorta tubular portion were measured by two experienced radiologists in consensus (S.J.C. and H.J.K.). AVA_CT_ was calculated by using the LVOT area measured on CT in the continuity equation with VTI at LVOT and transaortic flow:

$${\text{AVA}}_{{{\text{CT}}}} = {\text{LVOT}}_{{{\text{CT}}}} \times {\text{VTI}}_{{{\text{LVOT}}}} /{\text{VTI}}_{{{\text{Ao}}}}.$$

AVC was defined as a CT density of 130 Hounsfield units or greater confined to AV on non-enhanced cardiac gated images and measured using the methods suggested by Agatston et al.^30^. The AVC was measured using a commercially available software (Syngo.via Siemens Healthcare, Berlin, Germany). For stratification by sex, AVC_ratio_ was calculated by dividing AVC with sex-specific thresholds (Male, 2000; Female, 1250)^31^.

Systolic phase with largest AVA (20–30% RR) was selected and thick multiplanar reconstruction images were used to demarcate the tips of the aortic cusps for measuring AVA_plani_. To evaluate reliability of CT measurements, a third experienced radiologist (Y.A.) measured CT parameters in 100 randomly selected cases and interobserver agreement was determined. Observers were blinded to clinical data including echocardiography findings and operation records.

### Statistics

Continuous variables were expressed as mean ± standard deviation or median with IQR and categorical variables are presented as numbers and percentages. Interobserver agreement of CT findings was determined using a two-way random model ICC with consistency assumption. Comparison of AVA_echo_, AVA_CT_, and CT-derived AVA_plani_ was performed using Pearson correlations and Bland–Altman plots were graphed. One way ANOVA with post-hoc (Tukey) test or Kruskall–Wallis test and Chi-square test were used to compare baseline clinical and radiological findings among high-gradient severe AS, classic LF-LG AS, paradoxical LF-LG AS, and moderate AS groups. Bonferroni correction was applied to control the type I error for multiple comparison, and P-value 0.05/4 = 0.0125 was used for comparing the three groups. Student *t* test and Chi-square test were performed to compare two subgroups among the three groups. In LF-LG AS patients, clinical and CT findings for AVA_CT_ < 1.2 cm^2^ and AVA_CT_ ≥ 1.2 cm^2^ were compared using the Student *t* test and Chi-square test or Fisher’s exact test. For the stratification of risk factors for MACCE after AVR, cox proportional hazard models were used. Kaplan–Meier survival curves were drawn for statistically significant factors to predict MACCE. The 95% CIs were calculated and factors with *P* < 0.10 were included for multivariable cox regression analysis with enter method. To avoid multicollinearity, one of the aortic root parameters was included in the multivariable analysis among the CT parameters significantly associated with MACCE in univariate analysis. For BNP analysis, a continuous parameter was used and a cut-off of 700 pg/mL^32^ was set for outcome analysis using Kaplan–Meier curves^17^. P < 0.05 was considered statistically significant, except for multiple dependant variable analyses. Statistical analysis was performed using commercial software (SPSS, version 20; SPSS, Chicago, IL, USA).

## Supplementary Information

Supplementary Information.

## Data Availability

All data used during the current study are available from the corresponding author upon reasonable request.
