# Supplementary material for: Classification of severe aortic stenosis and outcomes after aortic valve replacement
Source: Sci Rep. 2022 May 7;12:7506. doi: 10.1038/s41598-022-11491-3 (PMC9079063; doi:10.1038/s41598-022-11491-3)
Supplement: Supplementary file 1 — Supplementary Information. [file 41598_2022_11491_MOESM1_ESM.docx]

**Classification of severe aortic stenosis and outcomes after aortic valve replacement**

**Supplementary Materials**

**Supplementary File 1.** Cardiac CT protocol

Unless contraindicated, 2.5 mg oral bisoprolol (Concor; Merck, Darmstadt, Germany) was administered to patients with heart rates >75 beats/min one hour prior to the CT scan for coronary artery evaluation. Before contrast material injection, single phase non-enhanced cardiac CT examination was performed in all patients using electrocardiogram (ECG) gating for AV calcium score calculation. After that, a bolus of 60–80 mL of nonionic, iodinated contrast material (Iomeron; Bracco Imaging SpA, Milan, Italy) was injected using a power injector (Stellant D; Medrad, Indianola, PA, USA) at 3.0 mL/s, followed by 40 mL of a 30:70 mixture of contrast and saline using the bolus tracking method (ascending aorta; trigger threshold level 100 HU; scan delay, 8 s). Retrospective ECG-gated scanning was performed with a 20% tube current modulation in lower phases (dose pulsing windows, 20%–70% of the R-R interval). In patients with arrhythmia, 0%–90% of the R-R interval was used. Tube voltage and the tube current-time product were adjusted for body size. The scan parameters were as follows: tube voltage, 80–120 kV; tube current, 160–360 mAs; pitch, 0.17–0.38; detector collimation, 64 × 0.6 mm and gantry rotation time, 280 ms. For preoperative evaluation of the aorta, most patients underwent CT of the thorax and abdomen as well as cardiac CT. Therefore, the DLP and the effective dose values were slightly higher compared to those used for routine cardiac CT scan. In all patients, the mean ± standard deviation dose-length product for cardiac CT scans was 1187.2 ± 547.7 mGy·cm and the mean effective dose was 16.6 ± 7.7 mSv.

**Supplementary Figure 1**. On CT, aortic valve (AV) is evaluated on the AV in-plane view which is parallel to the transverse plane of the aortic annulus. For evaluation of the aortic valve area using planimetry method, 5-10 mm slice thickness image is generated to delineate the tips of aortic cusps.


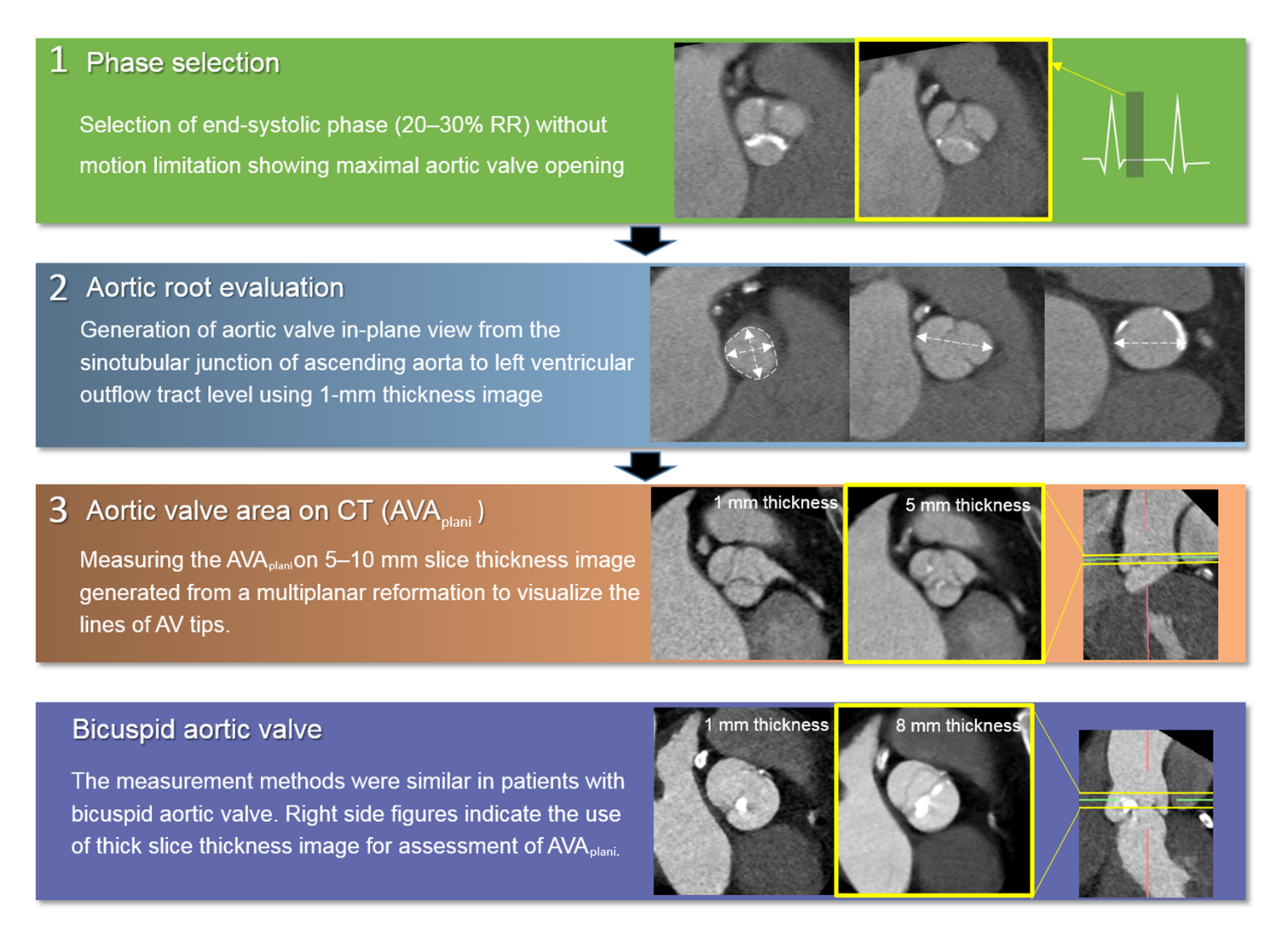


**Supplementary Figure 2**. Pearson correlation analysis result and Bland−Altman plot to comparison of AVA_plani_ and AVA_echo_


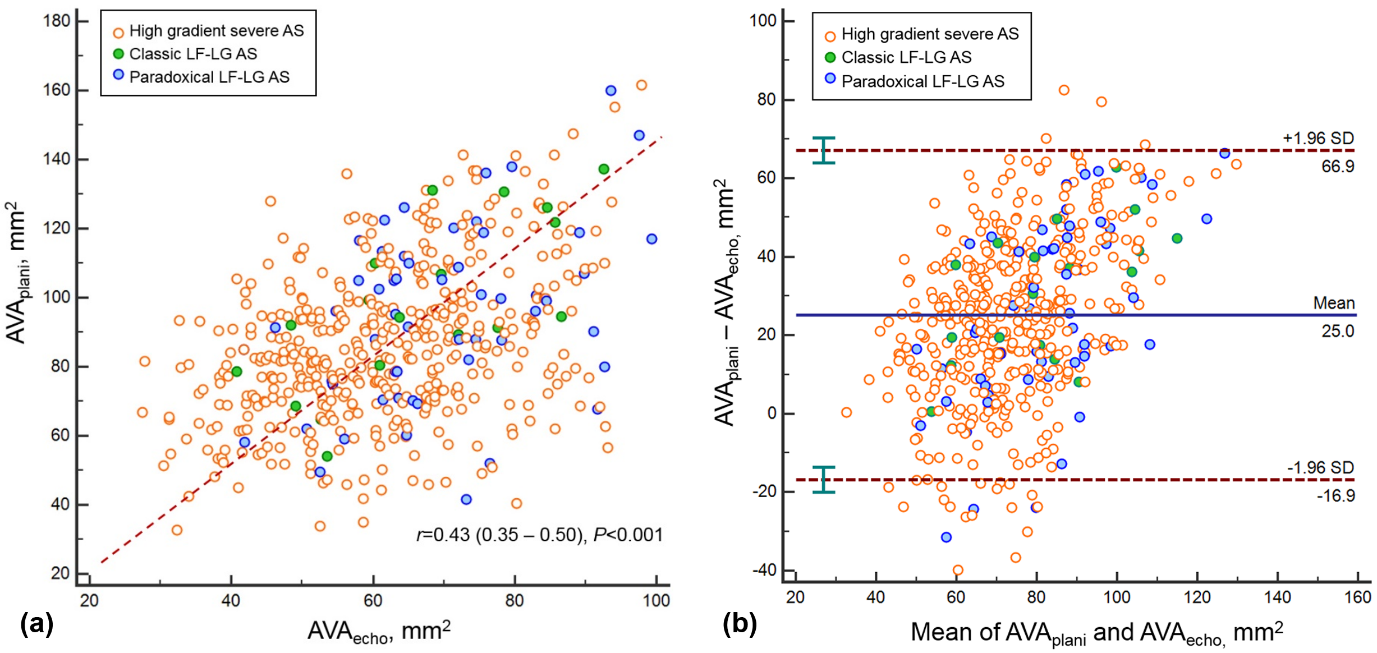


**Supplementary Figure 3. Comparison of aortic stenosis classification by echocardiography and cardiac CT**

Numbers in boxes and bars are absolute number of patients. AS, aortic stenosis; AVA, aortic valve area; LF-LG, low-flow and low-gradient.


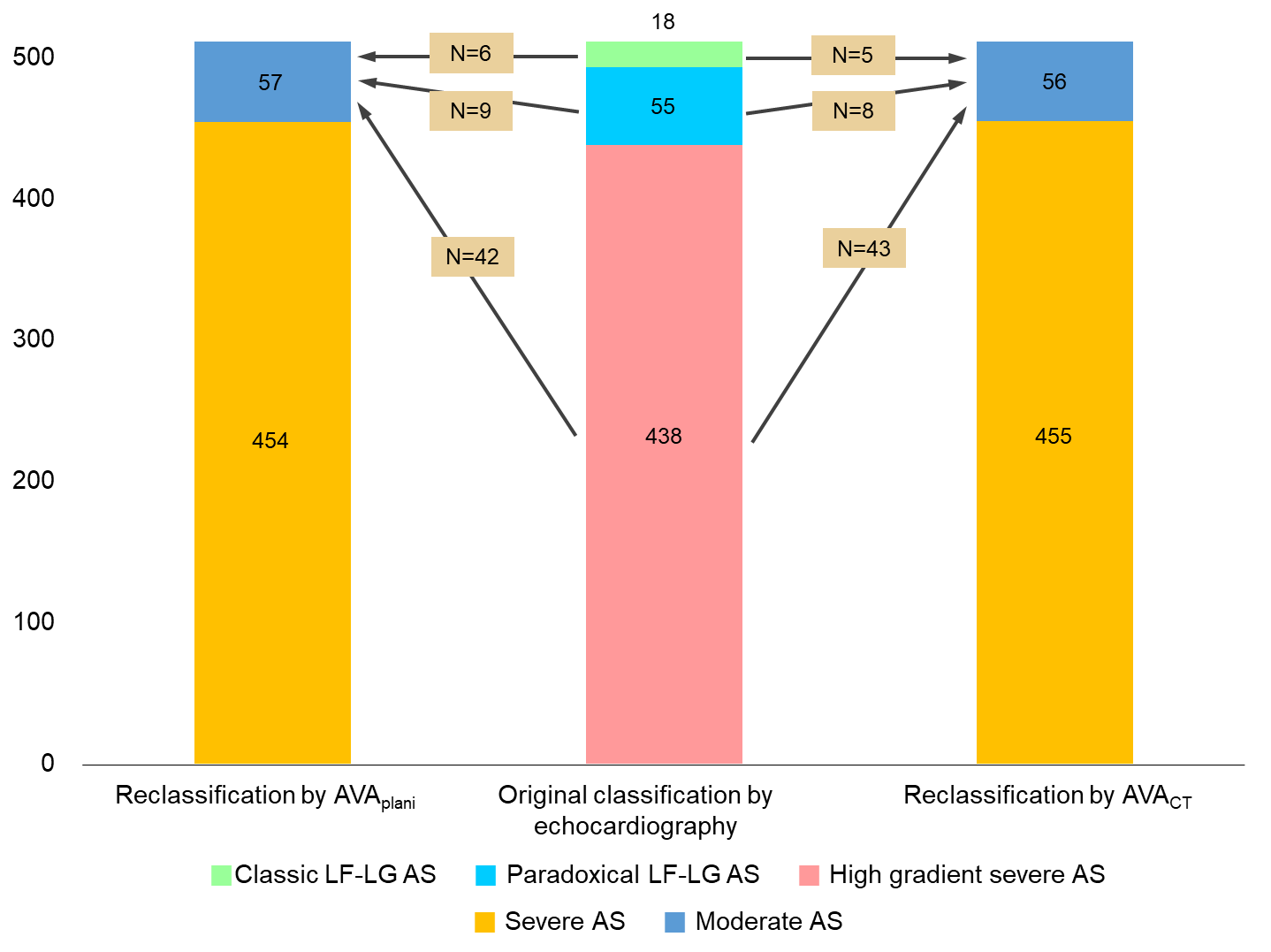


**Supplementary Table 1.** Patient characteristics (*n =* 511)

| Characteristic |  |
| --- | --- |
| Age, years | 66.9 ± 8.8 |
| Male | 280 (54.8) |
| Body surface area, m^2^ | 1.6 ± 0.2 |
| Hypertension | 273 (53.4) |
| Atrial fibrillation | 73 (14.3) |
| B-type natriuretic peptide*, pg/mL | 100.0 (43.0–287.0) |
| ln BNP | 4.8 ± 1.4 |
| Blood urea nitrogen, mg/dL | 18.2 ± 8.0 |
| Creatinine, mg/dL | 1.0 ± 0.9 |
| Echocardiography |  |
| LVEF, % | 59.7 ± 10.7 |
| Peak velocity, m/s | 4.9 ± 0.9 |
| Mean PG, mmHg | 61.0 ± 22.6 |
| LVMI, gm/ m^2^ | 134.8 ± 35.9 |
| Aortic valve VTI, cm | 122.2 ± 27.3 |
| LVOT VII, cm | 21.2 ± 4.2 |
| LVOT diameter, mm | 21.0 ± 1.5 |
| AVA_echo_, mm^2^ | 62.4 ± 14.9 |
| ESVI, mL/m^2^ | 28.6 ± 18.0 |
| EDVI, mL/m^2^ | 67.6 ± 24.6 |
| Systemic arterial compliance, mL/m^2^/mmHg | 0.8 ± 0.3 |
| Valvulo-arterial impedance, mmHg/mL/m^2^ | 5.2 ± 1.6 |
| Subgroups |  |
| Severe AS | 438 (85.7) |
| Classic LF-LG AS | 18 (3.5) |
| Paradoxical LF-LG AS | 55 (10.8) |
| CT findings |  |
| Valve morphology |  |
| Tricuspid | 246 (48.1) |
| Bicuspid with raphe | 126 (24.7) |
| Bicuspid without raphe | 139 (27.2) |
| AVC* | 2709.1 (1497.0–4033.6) |
| ln AVC* | 7.9 (7.3–8.3) |
| AVA_plani_, mm^2^ | 88.7 ± 24.0 |
| Calculated AVA_CT_ | 86.8 ± 23.2 |
| Aortic annulus |  |
| Circularity, % | 81.4 ± 7.4 |
| Mean diameter, mm | 25.0 ± 2.6 |
| Perimeter, mm | 79.8 ± 8.4 |
| Area, mm^2^ | 485.2 ± 102.8 |
| Sinus of Valsalva diameter, mm | 36.6 ± 4.6 |
| Sinotubular junction diameter, mm | 31.0 ± 4.7 |
| Ascending aorta tubular portion, mm | 40.6 ± 6.4 |
| Surgical valve size, mm | 22.1 ± 2.1 |
| Follow-up duration, year | 3.8 ± 1.6 |
| MACCE including cardiovascular death | 43 (8.4) |
| All-cause mortality | 71 (13.9) |

Note.–Data are numbers and percentages in parentheses, or mean and standard deviation.

*Data are median and interquartile range in parentheses

AS, aortic stenosis; AVA, aortic valve area; AVC, aortic valve calcium score; EDVI, end-diastolic volume index; ESVI, end-systolic volume index; LF-LG, low-flow and low-gradient; LVEF, left ventricular ejection fraction; LVMI, left ventricular mass index; LVOT, left ventricular outflow tract; MACCE, major adverse cardiac and cerebrovascular event; VTI, velocity time integral.

**Supplementary Table 2.** Interobserver agreements for CT parameters

| CT parameters | Intraclass correlation coefficient (*P*-value) |
| --- | --- |
| AVA_plani_ | 93.9 (<0.001) |
| Annulus maximal diameter | 95.9 (<0.001) |
| Annulus maximal diameter | 93.9 (<0.001) |
| Annulus perimeter | 96.7 (<0.001) |
| Annulus area | 96.1 (<0.001) |
| Sinus of Valsalva maximal diameter | 97.0 (<0.001) |
| Sinotubular junction diameter | 89.2 (<0.001) |
| Ascending aorta tubular portion | 98.6 (<0.001) |

AVA_CT_, aortic valve area measured on computed tomography (CT) images
